# Learning to Unveil: Tackling Implicit Bias in Pain Recognition Through Education

**DOI:** 10.1002/ejp.70281

**Published:** 2026-04-25

**Authors:** Arianna Bagnis, Ilenia Ceccarelli, Franco Stella, Katia Mattarozzi

**Affiliations:** ^1^ Department of Medical and Surgical Sciences University of Bologna Bologna Italy

**Keywords:** education, facial trustworthiness, implicit bias, medical students, pain recognition, race, simulation training

## Abstract

**Background:**

Independent studies demonstrate that racial biases and inferences from facial appearance impact healthcare decisions, especially in pain recognition and treatment, with such biases already detectable among medical students. To address this issue, the present research evaluated the effectiveness of a multifaceted evidence‐based educational intervention aimed at mitigating implicit biases by increasing students' knowledge and awareness of these factors in clinical settings and fostering strategies for equitable pain management.

**Method:**

A total of 100 medical students were randomly assigned to an experimental or a control group. Both groups completed a pain recognition task twice, evaluating perceived pain intensity and the likelihood of recommending treatment. Between sessions, the experimental group took part in a brief educational intervention combining theoretical input on implicit biases in pain assessment, evidence from empirical studies, and applied reflection on clinical scenarios, whereas the control group received the same lesson after completing the study.

**Results:**

The findings reveal that repeated exposure to the pain recognition task influenced responses in both groups, suggesting a task‐related learning effect. The educational intervention significantly improved response times, pain intensity ratings, and treatment recommendations across stimuli categories, irrespective of race or facial trustworthiness.

**Conclusions:**

This suggests that the intervention heightened students' sensitivity to pain‐related cues and encouraged a re‐evaluation of clinical judgements. These results underscore the value of targeted educational initiatives in addressing disparities in pain recognition and treatment driven by facial cues, providing evidence that even brief interventions may contribute to mitigating implicit biases and support more equitable healthcare decision‐making.

**Significance:**

This study demonstrates the effectiveness of a brief, evidence‐based educational intervention in reducing implicit racial biases in pain recognition among medical students. By enhancing students' sensitivity to pain‐related cues, the intervention holds promise for improving equitable healthcare practices and reducing bias‐driven disparities in pain management.

## 1 Introduction

The subjective nature of pain makes its recognition and assessment in others challenging, especially considering that healthcare professionals are not immune to implicit biases, such as racial stereotypes and assumptions about pain tolerance (Ruben et al. 2018). These biases can distort pain judgements, contributing to healthcare disparities and leading to inadequate treatment for some individuals (Green et al. 2006; Telusca et al. 2022).

Racial biases among healthcare providers and their impact on treatment decisions have been studied extensively (Ashton‐James et al. 2023; Webb Hooper et al. 2020; Lee et al. 2019; Meghani et al. 2012; Morden et al. 2021). Studies conducted in clinical settings alarmingly indicate that pain experienced by Black compared to White patients is often underestimated and undertreated (Anderson et al. 2009; Green et al. 2003; Staton et al. 2007).

Independent studies demonstrate that inferences from facial appearance impact healthcare decisions as well (Bagnis et al. 2020; Colonnello, Mattarozzi, and Russo 2019; Mattarozzi et al. 2017; Schäfer et al. 2016; Todorov et al. 2015). Perceived untrustworthiness from facial appearance has been associated with a lower likelihood of receiving priority treatment in emergency units (Bagnis et al. 2020). Facial trustworthiness impacts both doctors' judgments about the authenticity of patients' pain expression (Schäfer et al. 2016) and emotion recognition, with untrustworthy‐looking faces leading to reduced accuracy and speed in recognizing emotion compared to trustworthy‐looking faces (Colonnello, Russo, and Mattarozzi 2019).

Implicit biases are already present among medical students (Haider et al. 2011). Students believing in false biological racial differences tend to underestimate Black patients' pain and make poorer treatment recommendations (Hoffman et al. 2016). Similarly, facial trustworthiness acts as a source of implicit bias, with medical students showing slower and less accurate emotion recognition for untrustworthy‐looking faces (Colonnello, Mattarozzi, and Russo 2019). Recent research highlights that the interaction between race and facial trustworthiness impacts the ability to recognize painful expressions in medical students (Ceccarelli et al. 2024).

Although biases in pain recognition are prevalent, their negative effects can be mitigated through increased awareness and targeted training. Implicit bias interventions have been shown to improve medical students' racial attitudes, including reductions in Black‐White IAT scores (Ruben and Saks 2020; van Ryn et al. 2015). Additionally, facial appearance‐based bias can be mitigated by activating positive professional caregiving schemas in medical students (Colonnello, Mattarozzi, and Russo 2019). Computer‐based simulation tools are increasingly used in health professions education to create experiential learning opportunities, allowing learners to engage with realistic clinical scenarios in a controlled environment (Elcokany et al. 2021; Ward et al. 2019).

Building on these findings, this study evaluates the effectiveness of a multifaceted evidence‐based educational intervention incorporating simulation‐based training, designed to mitigate implicit race‐ and facial trustworthiness–based biases by increasing knowledge and awareness and promoting equitable pain assessment. Specifically, the pain recognition task was embedded within a simulation‐based educational framework, serving both as an outcome measure and as an experiential learning tool.

We hypothesize that students receiving the intervention will demonstrate less biased pain perception, showing no differences in response time and perceived pain intensity, based on the stimulus race or facial trustworthiness, and will provide more equitable treatment recommendations than controls.

## 2 Methods

### 2.1 Participants

All participants were second‐year medical students (cohort 2022/2023) enrolled in a mandatory Cognitive Psychology course, which ensured that the sample represented the entire class and no additional recruitment was needed. As part of this class, a multifaceted educational intervention has been integrated to enhance their understanding of cognitive processes and their application in medical practice. Participation in the educational activity and completion of the questionnaires were presented as part of their regular course engagement. To ensure anonymity, students were instructed to create an alphanumeric code based on simple guidelines to identify their responses without revealing their identities. All participants signed an informed consent form prior to the study. In line with standard practice for research involving implicit measures, the consent form described the session as involving cognitive psychology tasks, without disclosing the specific research question or the experimental variables and their manipulation, in order to prevent response bias. Participants were fully debriefed about the research question and study aims at the end of their participation, as described in the educational intervention section. Although participation in the educational activity was mandatory as part of the course content, students were explicitly informed that the use of their anonymized data for research purposes was entirely voluntary. They could refuse consent for data use without any consequences. This choice was made anonymously, and the teacher had no access to information identifying which students declined participation, ensuring that participation in the research component was entirely voluntary. No students chose to opt out.

Based on the power analysis conducted using G*Power (ANOVA: Repeated measures, within‐between interactions; (Faul et al. 2007)), a sample size of 90 participants was required to achieve an effect size of f = 0.10, with two groups, eight measurements, a correlation among repeated measures of 0.5, an alpha level of 0.05, and a power of 0.80. Response time was used as the reference variable for this calculation, as it was considered a conservative choice given its sensitivity to implicit processes and the typically smaller effect sizes reported for this measure in the literature (Tajeu et al. 2022). Accordingly, 100 2^nd^ year medical students were recruited from the University of Bologna.

### 2.2 Procedure

The experimental procedure is in accordance with the Helsinki Declaration (1975, 1983) and was approved by the Institutional Review Board of the University of Bologna.

**FIGURE 1:**
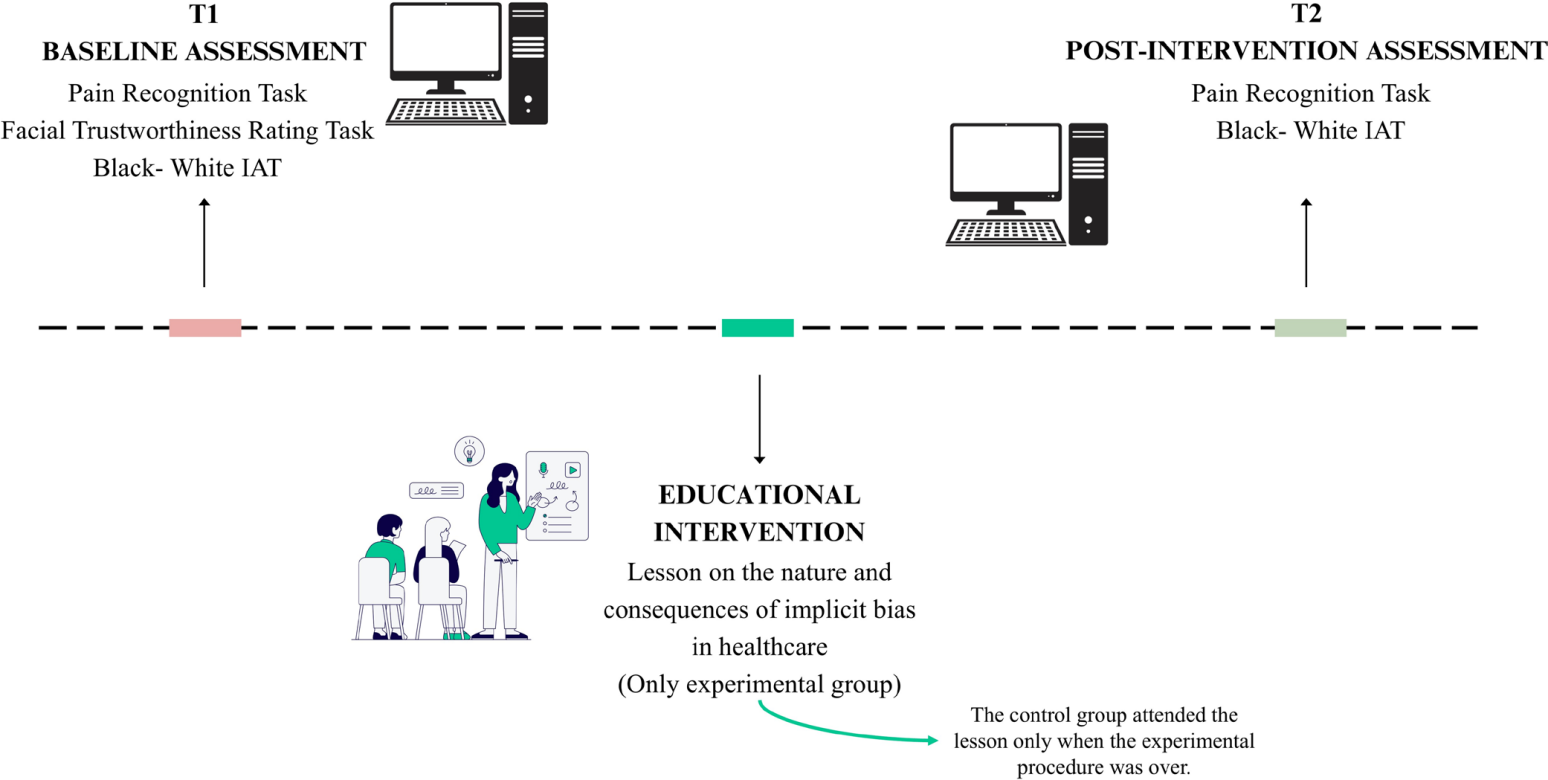
Experimental procedure.

The research has been conducted in three main phases: baseline assessment (T1), educational intervention, and post‐intervention assessment (T2). The procedure for each phase is shown in Figure 1.

**FIGURE 2:**
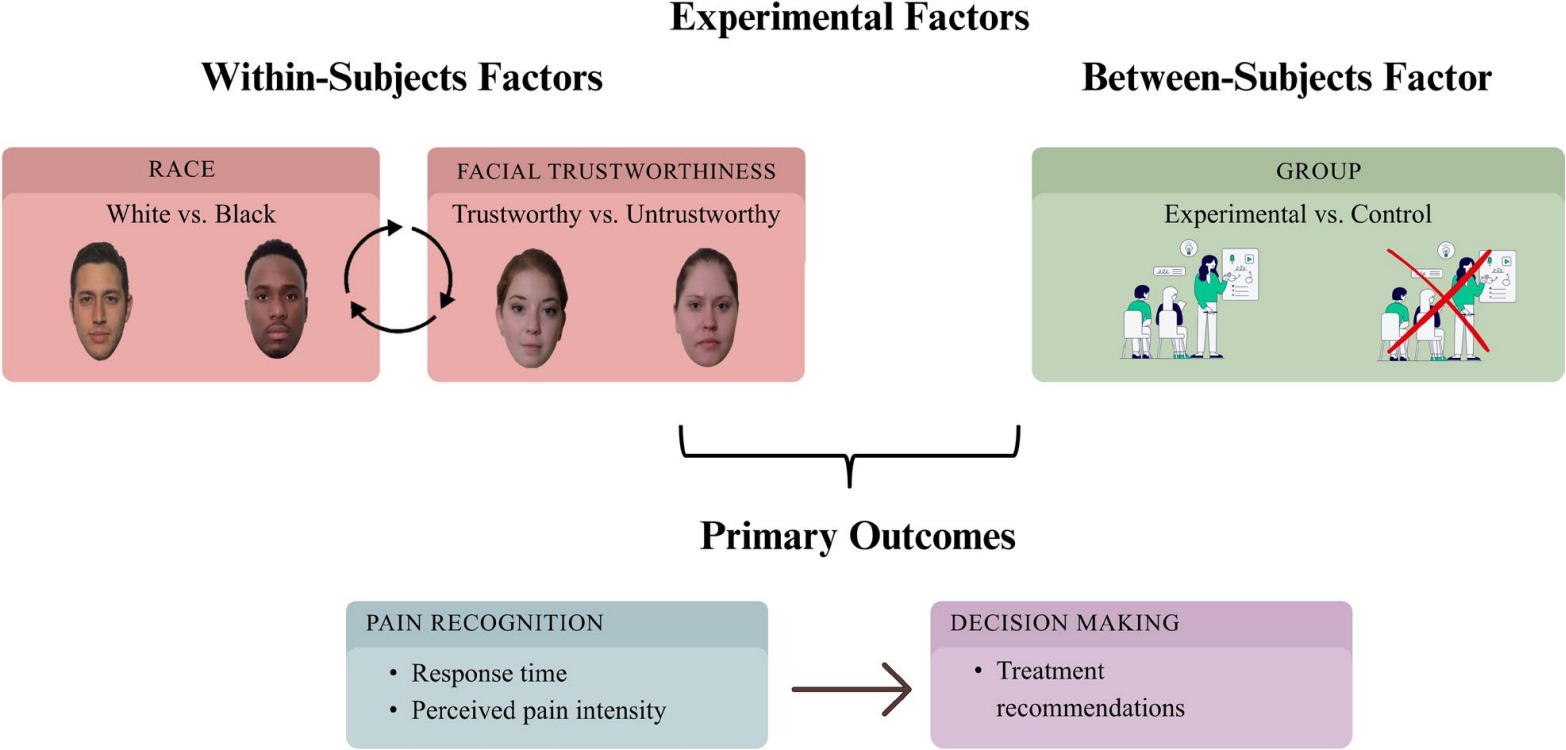
Experimental factors and primary outcomes.

The study employed a (2 × 2 × 2) × 2 mixed model design. The within‐subjects factors were race (White vs. Black), facial trustworthiness (Trustworthy vs. Untrustworthy), and time (Pre vs. Post educational intervention). The between‐subjects factor was group (Experimental vs. Control). The three primary outcomes (see below for more details) were response time (ms), perceived pain intensity, and treatment recommendation, all measured at both T1 and T2. See Figure 2 (please note that factor “Time” is not shown to keep the figure clearer). Participants were randomly assigned to either the experimental or control group using a computerized simple randomization procedure without allocation restrictions. The groups were kept in separate spaces and participated in the session sequentially, with no opportunity to meet or interact at any point during the experimental session.

### 2.3 Pain Recognition Task

Once participants entered the lab at the University, they were asked to sit in front of the computer screen (monitor dimension: 22 × 49.2 × 37.6 cm; visual angle: 178°) where the task would be administered. They received oral and written instructions, and two practice trials were given to become familiar with the task, which was composed of 16 trials in total. Each trial was preceded by a central fixation cross, a small cross‐shaped marker displayed at the centre of the screen for a fixed duration, used to direct participants' gaze to the centre of the display and to standardize their visual attention before the onset of each stimulus. The video clips presentation order was pseudorandomized, controlling for facial trustworthiness and race. The total duration of the task was ~20 min. The stimuli consisted of 16 video clips, each 6 s long and presented at 25 frames per second. Each video began with a neutral facial expression that gradually changed into a full‐intensity painful expression. Participants were instructed to press the spacebar as soon as they perceived the facial expression as painful (response time). Afterward, they rated the intensity of the pain displayed (perceived pain intensity) and reported the likelihood of recommending treatment if they were the doctor in charge of treating them (treatment recommendation). Participants evaluated perceived pain intensity and treatment recommendation based on a still frame from the video at the moment they perceived pain, without watching the video to the end. This methodological choice ensures that judgments are made precisely at the point of pain detection, standardizing the phenomenological quality of the stimulus across conditions (e.g., race or trustworthiness). This approach allows for a more accurate assessment of how race and facial trustworthiness impact response times and, then, pain intensity judgments and treatment recommendation.

Data were collected on these three primary outcomes during both T1 and T2. Response time in milliseconds (ms), perceived pain intensity and recommendation of treatment on Likert scales (0 = not at all intense to 10 = extremely intense; 0 = not at all likely to 10 = extremely likely, respectively) were recorded. For stimulus presentation and response data collection, E‐Prime 2 software (Psychology software tools, Pittsburgh, USA) was used. The task is based on an Emotion Recognition Task paradigm previously validated in multiple peer‐reviewed studies (Bagnis et al. 2024; Colonnello et al. 2021; Colonnello, Mattarozzi, and Russo 2019; Colonnello, Russo, and Mattarozzi 2019), and was pre‐tested internally within the research group prior to data collection to ensure correct functioning and timing.

The clips were designed to ensure a balanced representation of race and facial trustworthiness: (i) White Faces: 8 video clips (4 trustworthy faces: 2 females, 2 males; 4 untrustworthy faces: 2 females, 2 males); (ii) Black Faces: 8 video clips (4 trustworthy faces: 2 females, 2 males; 4 untrustworthy faces: 2 females, 2 males). To create the video clips, we selected trustworthy‐looking and untrustworthy‐looking adult faces from the Delaware Pain Database (Mende‐Siedlecki et al. 2020), ensuring no differences on trustworthiness and painful expression ratings across races in the source database. The stimuli for the T2 task were different from those used in the T1 task, but they were matched to the T1 stimuli. For more details on the selection process, see Supporting Information. Four additional identities were included for practice trials. For each selected identity, we used images depicting both neutral and painful facial expressions. Each image was morphed using FantaMorph software to create sequences with increasing painful intensity, where the neutral and full painful faces represent the first and last frames, respectively. This morphing technique enhances the ecological validity of the study by simulating the natural variability and complexity of emotional expressions as they occur in real life.

### 2.4 Facial Trustworthiness Rating Task

Following the pain recognition task, participants completed a face rating task to control how they evaluated the stimuli according to the facial trustworthiness level (manipulation check). Specifically, this task required participants to carefully observe each face and respond based on their first impression. The stimuli included 32 faces, of which 16 were used in the pain recognition task and 16 were new (fillers). Participants rated the trustworthiness of the face on a Likert scale from 0 (not at all) to 10 (extremely). The task was administered using the Qualtrics platform.

### 2.5 Black‐White Implicit Association Test (IAT)

Participants completed the computer‐based Implicit Association Test (IAT) at both T1 and T2 to assess individual differences in implicit racial bias (Greenwald et al. 1998). The test consisted of seven blocks and evaluated the strength of automatic associations between two social groups (White and Black) and two categories (positive and negative). During each trial, participants were instructed to quickly associate Black and White faces with either positive words (e.g., happiness) or negative words (e.g., agony). The strength of these associations was determined by comparing response times for Black/positive and White/negative pairings with those for Black/negative and White/positive pairings. A *d*‐value greater than zero indicates an implicit preference for White individuals over Black individuals (Greenwald 2003; Greenwald et al. 1998; Greenwald and Banaji 1995).

### 2.6 Educational Intervention

Participants attended a multifaceted educational intervention of 3 h focused on pain recognition, and on the nature and consequences of implicit biases in healthcare. The educational intervention incorporated simulation‐based training elements and was designed from existing literature on pain, face perception, racial bias, social inferences from facial appearance, and the role of awareness on these biases (Devine et al. 2012; Farah et al. 1998; Fitzgerald et al. 2019; Raja et al. 2020; Strand et al. 2021; Sukhera and Watling 2018; Telusca et al. 2022; Todorov et al. 2015). In line with current reporting recommendations for simulation‐based research (e.g., INSPIRE guidelines), the intervention can be conceptualized as a simulation‐based educational activity combining experiential and reflective learning components. Specifically, the pain recognition task functioned as an interactive computer‐based simulation, in which participants engaged with standardized, dynamic facial stimuli that mimicked clinically relevant cues of pain expression. This simulation component was used both as an assessment tool and as an experiential learning activity aimed at increasing awareness of perceptual and cognitive biases in pain recognition. The multifaceted approach started with experiential learning through this simulation‐based task, followed by a structured lecture and guided reflection encompassing the following key areas:

- Importance of Faces as Social Stimuli: the lecture outlined the evolutionary significance of facial expressions, emphasizing their roles as salient social signals. Students learned about the communicative value of these expressions in fostering social connections and the informative nature of facial cues, even in the absence of overt expressions. This section detailed how physical facial features convey aspects of identity, race, and inferences about personality traits, all of which influence decision‐making and behaviour, particularly in situations where information is limited.
- Psychological Underpinnings of Biases: an evidence‐based framework was provided to explore the psychological mechanisms underlying biases related to race and facial appearance. Discussions addressed how automatic inferences can lead to inferences regarding individuals based on their physical characteristics.
- Understanding Pain as an Emotional Experience: students were educated on pain as an unpleasant emotional response, which serves as both an intra‐ and interpersonal signal. This segment emphasized the complexity of pain recognition and its implications for interpersonal interactions and patient care. Additionally, it focused on the factors influencing the speed and accuracy of pain recognition in others. It included definitions and examples of implicit biases, illustrating how such biases can affect judgments and decisions in clinical settings. Participants also learned how race and inferences from facial appearance such as familiarity and trustworthiness can influence pain recognition processes.

Finally, several evidence‐based strategies (Devine et al. 2012) were introduced to help students recognize and counteract implicit biases in clinical practice. These strategies included Stereotype Replacement, which teaches students to identify and replace stereotypical responses with more appropriate reactions. Counterstereotype Imaging encourages visualization of individuals who defy prevalent stereotypes (e.g., Black individuals or those with unfamiliar and untrustworthy faces), promoting a more nuanced understanding. The approach of Individuation fosters the recognition of individuals' unique backgrounds, allowing students to see them as distinct persons rather than mere stereotypes. Additionally, Perspective Taking cultivates the ability to adopt the viewpoints of others, enhancing empathy and understanding of diverse experiences. After the pain recognition task had been completed for the second time, a session was organized to present the results obtained by the group of students, reinforcing the findings with corroborating literature. This session aimed to highlight the complexity of the pain recognition process in others and to emphasize the students' vulnerabilities to biases related to race and facial appearance. By examining their own performance in light of established research, students gained deeper insights into how these biases can influence clinical judgements.

To evaluate the overall impact of the training, a questionnaire was administered via computer at the end of the session, using a 4‐point Likert scale ranging “Definitely No” to “Definitely Yes” to assess the clarity, interest, and satisfaction of the students with the training they had received.

## 3 Statistical Analysis

Differences between the two groups in terms of sex distribution, age, and IAT score were calculated using *χ* ^*2*^‐tests and *t*‐tests as appropriate.

Outliers were identified based on participants' average values across primary outcomes (response times, pain intensity ratings, treatment recommendations). Specifically, any case with a mean score exceeding ±3 standard deviations from the group mean on any of these variables was flagged for exclusion. We also excluded participants who showed inconsistent or random‐like patterns across trials (e.g., flat responses or alternating extreme values), which we considered indicative of low engagement or misunderstanding of the task.

Statistical analyses were conducted to ensure the validity of the experimental procedures and to evaluate the impact of the educational intervention: (1) A facial trustworthiness check was performed on the face rating data (T1) to confirm that participants rated trustworthy faces as significantly more trustworthy than untrustworthy faces, ensuring the validity of the facial trustworthiness manipulation; (2) Baseline equivalence between the experimental and control groups was assessed using statistical tests on all three primary outcomes prior to the intervention (T1), confirming that there were no significant differences between groups at the outset; (3) The main analyses evaluated the effect of the educational intervention on the three primary outcomes. Here, the IAT score was included as a covariate to isolate the effect of the educational intervention from individual differences in implicit bias, aligning with the study's focus on evaluating the intervention's efficacy independently of pre‐existing bias levels.

As such, a series of mixed‐model Analyses of Variance (ANOVAs) and Analyses of Covariance (ANCOVAs) were conducted, examining the main effects and interactions of Race, Facial trustworthiness, Time, and Group. Post hoc Bonferroni‐corrected comparisons were performed to explore interactions and further interpret the findings. Effect sizes were calculated using partial eta squared. The alpha level for all analyses was set to *p* < 0.05. All the analyses were run using SPSS version 25.0 (Chicago, IL).

The results of the facial trustworthiness check (on T1 face rating data) and the statistical tests assessing baseline equivalence between groups on all primary outcomes are reported in the Supporting Information.

## 4 Results

### 4.1 Descriptive Statistics

The 100 participants were evenly divided between experimental and control groups. All participants self‐identified as White, which reflects the demographic composition of the student population in this academic context.

**FIGURE 3:**
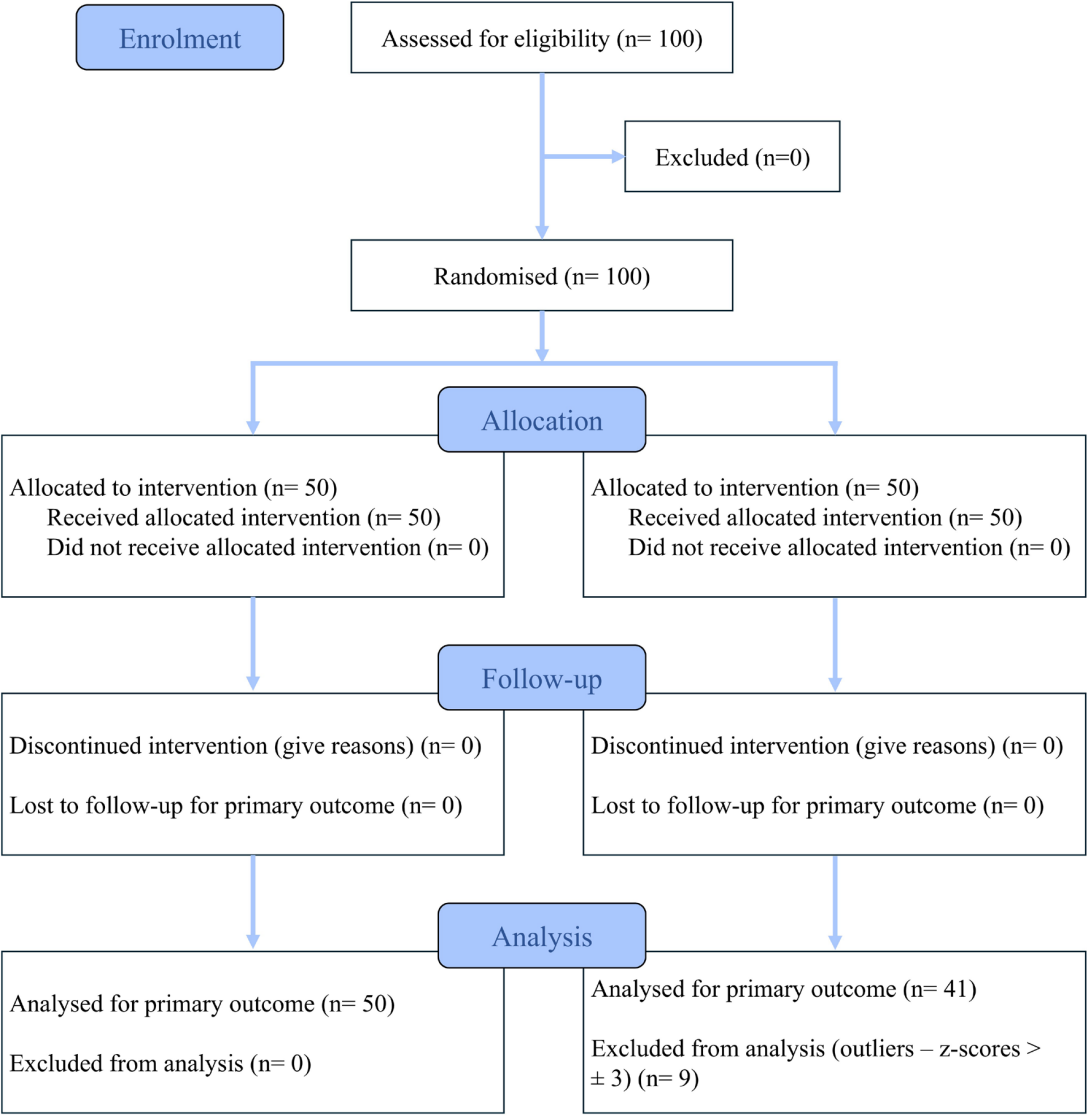
CONSORT flow diagram.

Nine participants were excluded from the analyses as outliers (*z*‐scores > ±3), resulting in a final sample of 91 participants (mean age = 20.42, SD = 0.95; 33 males, 58 females), with 50 in the experimental group and 41 in the control group (see Figure 3).

Due to a technical issue, the IAT score for one subject is missing. Therefore, for the ANCOVAs where the IAT is included as a covariate, the sample size is 90 participants (mean IAT score = 0.69, SD = 0.39). The groups did not differ in age, sex, or IAT score. Specifically, for age, the experimental group (M = 20.48, SD = 0.86) and the control group (M = 20.34, SD = 1.06) were not significantly different, *t*(89) = 0.69, *p* = 0.25. Regarding sex, the experimental group included 34 females and 16 males, while the control group included 24 females and 17 males, with no significant difference, *χ* ^*2*^ = 0.87, *p* = 0.39. For the IAT score, the experimental group (M = 0.70, SD = 0.35) and the control group (M = 0.68, SD = 0.42) also did not differ significantly, *t*(88) = 0.27, *p* = 0.39.

The results of the questionnaire highlighted a generally positive response from students regarding the training session. Participants reported a high level of clarity in the content delivered, with the majority (98.9% of students responded with “Definitely Yes” or “Mostly Yes”) indicating that the teacher presented the topics clearly. Interest in the subject matter was also evident, as many students (97.9% of students responded with “Definitely Yes” or “Mostly Yes”) expressed genuine engagement with the topics covered during the session. Additionally, overall satisfaction was notably high (100% of students responded with “Definitely Yes” or “Mostly Yes”), with participants affirming that the training met or exceeded their expectations. These findings underscore the effectiveness of the session in delivering clear, engaging, and satisfactory learning experiences.

## 5 Main Analyses

### 5.1 Response Time

The mixed ANCOVA revealed a significant time × race interaction (F(1,87) = 11.30, *p* = 0.001, ηp^2^ = 0.11), with medical students being faster at perceiving pain during T2 compared to T1 only when the pain was expressed by Black stimuli (T1: *M* = 4312.06 ms, SE = 91.03; T2: *M* = 3852.07 ms, SE = 122.52; *p* < 0.001), whereas no difference in response times was observed between T1 and T2 for White stimuli (T1: *M* = 4023.11 ms, SE = 96.24; T2: *M* = 3971.90 ms, SE = 121.66; *p* = 0.62).

**FIGURE 4:**
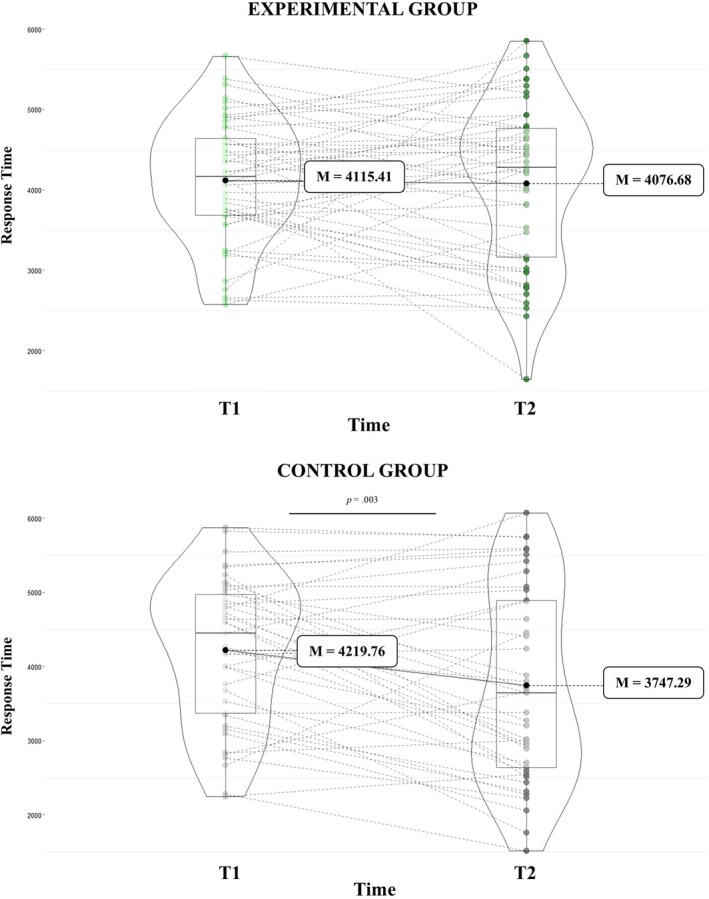
Response times across time points (T1 and T2) in control and experimental groups.

Interestingly, a significant group × time interaction (*F*_(1,87)_ = 4.39, *p* = 0.04, *η*_*p*_^*2*^ = 0.05) emerged. Medical students in the control group were faster at perceiving pain during T2 compared to T1 (T1: *M* = 4219.76 ms, SE = 136.03; T2: *M* = 3747.29 ms, SE = 178.02; *p* = 0.003). In contrast, no difference in response times was observed between T1 and T2 in the experimental group (T1: *M* = 4115.41 ms, SE = 124.42; T2: *M* = 4076.68 ms, SE = 162.83; *p* = 0.78). See Figure 4. No other significant main effects or interactions emerged.

### 5.2 Perceived Pain Intensity

The mixed ANCOVA revealed a significant main effect of facial trustworthiness (*F*_(1,87)_ = 11.91, *p* < 0.001, *η*_*p*_^*2*^ = 0.12), with untrustworthy‐looking faces (*M* = 5.88, SE = 0.10) being evaluated as expressing more pain than trustworthy‐looking faces (*M* = 5.51, SE = 0.11; *p* < 0.001).

Again, a significant time × race interaction (*F*_(1,87)_ = 19.93, *p* < 0.001, *η*_*p*_^*2*^ = 0.19) emerged, with Black faces (T1: *M* = 5.18, SE = 0.12; T2: *M* = 6.03, SE = 0.11; *p* < 0.001) being judged as more in pain during T2 compared to T1, whereas no difference was observed between T1 and T2 for White faces (T1: *M* = 5.73, SE = 0.12; T2: *M* = 5.84, SE = 0.12; *p* = 0.35). Additionally, the significant three‐way interaction of time × race × facial trustworthiness (*F*_(1,87)_ = 9.10, *p* = 0.002, *η*_*p*_^*2*^ = 0.10) allowed for further detailing of the observed differences, providing deeper insights into how these factors interact to influence pain judgements. Specifically, when the faces appeared trustworthy‐looking, pain intensity ratings increased at T2 compared to T1 for both White (T1: *M* = 5.63, SE = 0.14; T2: *M* = 6.19, SE = 0.13; *p* < 0.001) and Black (T1: *M* = 4.81, SE = 0.13; T2: *M* = 5.41, SE = 0.14; *p* < 0.001) faces. However, when the faces appeared untrustworthy‐looking, this increase was observed only for Black faces (T1: *M* = 5.56, SE = 0.13; T2: *M* = 6.65, SE = 0.10; *p* < 0.001). For untrustworthy‐looking White faces, the pattern reversed, with pain intensity ratings decreasing at T2 compared to T1 (T1: *M* = 5.82, SE = 0.13; T2: *M* = 5.49, SE = 0.14; *p* = 0.05).

**FIGURE 5:**
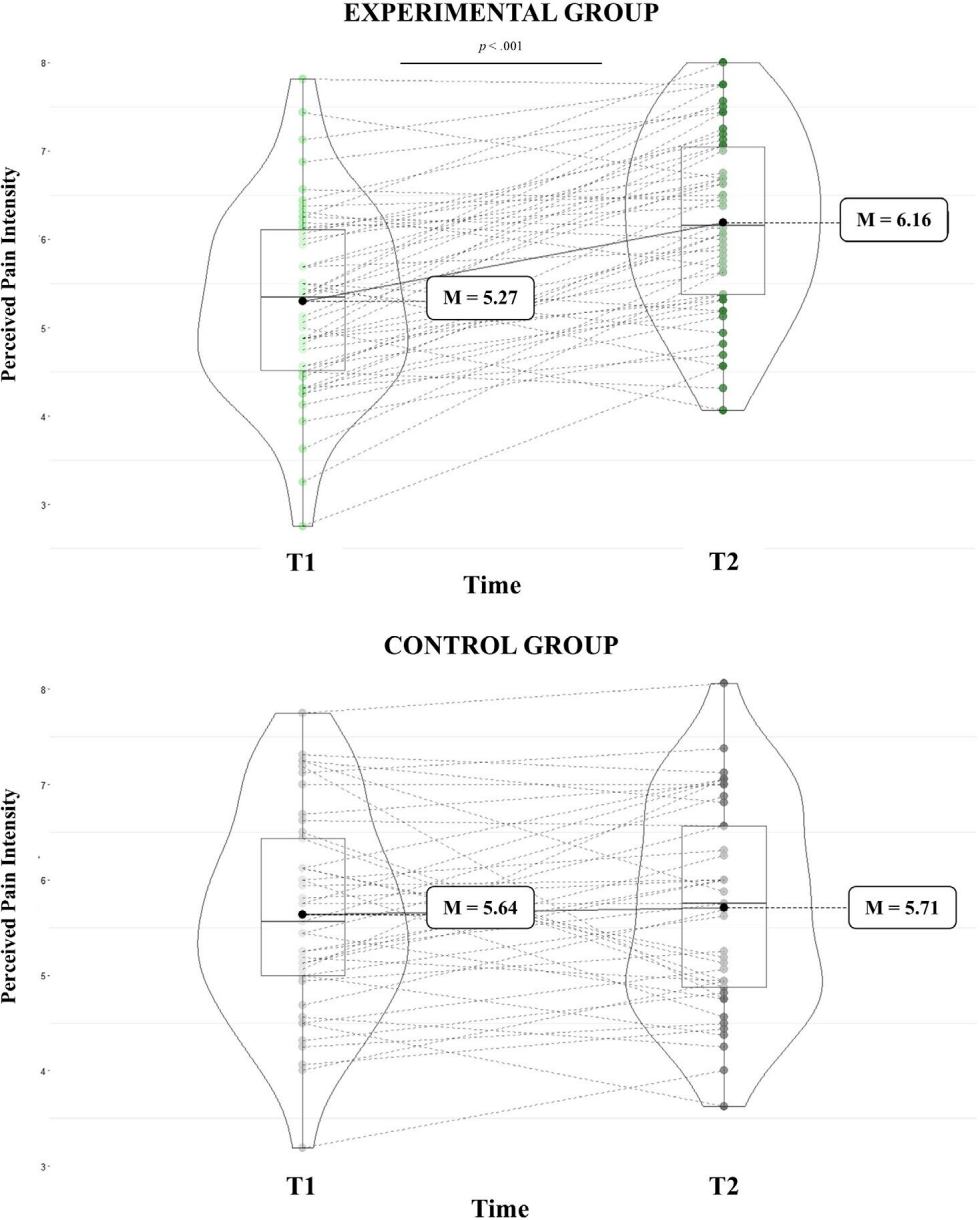
Perceived pain intensity across time points (T1 and T2) in control and experimental groups.

Interestingly, a significant group × time interaction was also found (*F*_(1,87)_ = 16.28, *p* < 0.001, *η*_*p*_^*2*^ = 0.16). Medical students in the experimental group perceived pain as more intense during the second task compared to the first (T1: *M* = 5.27, SE = 0.15; T2: *M* = 6.16, SE = 0.15; *p* < 0.001). In contrast, no difference in perceived pain intensity was observed between T1 and T2 in the control group (T1: *M* = 5.64, SE = 0.17; T2: *M* = 5.71, SE = 0.16; *p* = 0.61). See Figure 5. No other significant main effects or interactions emerged.

### 5.3 Treatment Recommendation

The mixed ANCOVA revealed a significant main effect of facial trustworthiness (*F*_(1,87)_ = 8.62, *p* = 0.004, *η*_*p*_^*2*^ = 0.09), with untrustworthy‐looking faces (*M* = 5.43, SE = 0.13) being more likely to receive therapy than trustworthy‐looking faces (*M* = 5.05, SE = 0.13; *p* < 0.001), and a significant main effect of time (*F*_(1,88)_ = 4.36, *p* = 0.04, *η*_*p*_^*2*^ = 0.05), with a higher likelihood of treatment recommendations in T2 (*M* = 5.47, SE = 0.14) than in T1 (*M* = 5.01, SE = 0.14; *p* < 0.001).

Again, a significant time × race interaction (*F*_(1,87)_ = 7.07, *p* = 0.009, *η*_*p*_^2^ = 0.07) emerged, with Black faces (T1: *M* = 4.80, SE = 0.15; T2: *M* = 5.59, SE = 0.14; *p* < 0.001) being more likely to receive therapy during T2 compared to T1, whereas no difference was observed between T1 and T2 for White stimuli (T1: *M* = 5.22, SE = 0.15; T2: *M* = 5.35, SE = 0.15; *p* = 0.37). Mirroring the results on perceived pain intensity, the three‐way interaction of time × race × facial trustworthiness (*F*_(1,87)_ = 9.55, *p* = 0.003, *η*_*p*_^*2*^ = 0.10) was found to be significant. When the faces appeared trustworthy‐looking, treatment recommendations increased at T2 compared to T1 for both White (T1: *M* = 5.09, SE = 0.16; T2: *M* = 5.75, SE = 0.16; *p* < 0.001) and Black (T1: *M* = 4.40, SE = 0.16; T2: *M* = 4.96, SE = 0.17; *p* = 0.002) faces. However, when the faces appeared untrustworthy‐looking, this increase was observed only for Black faces (T1: *M* = 5.20, SE = 0.17; T2: *M* = 6.13, SE = 0.13; *p* < 0.001). For untrustworthy‐looking White faces, the pattern reversed, with treatment recommendations decreasing at T2 compared to T1 (T1: *M* = 5.35, SE = 0.16; T2: *M* = 4.96, SE = 0.16; *p* = 0.03).

**FIGURE 6:**
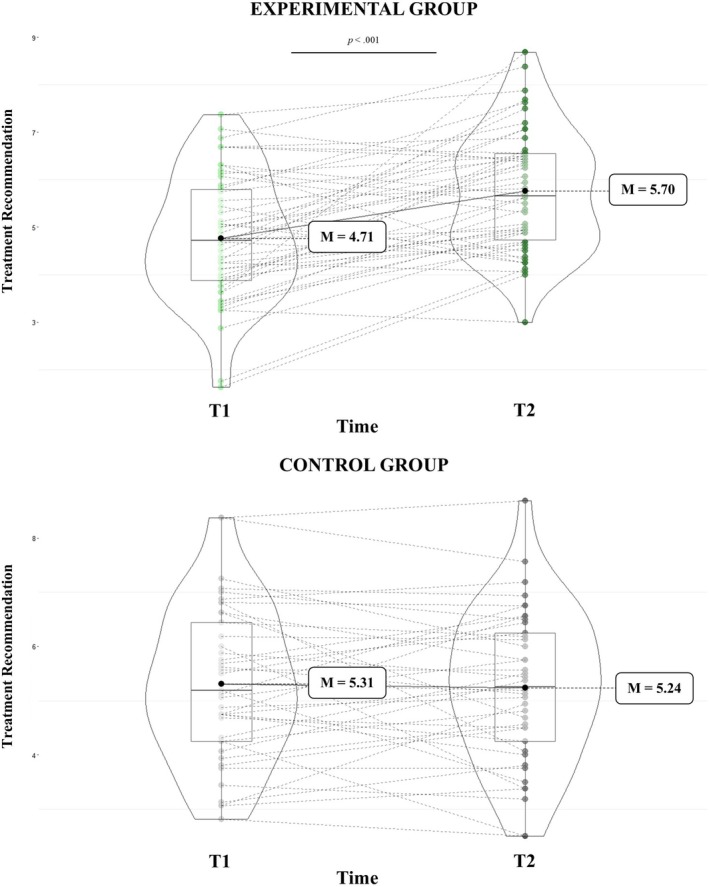
Treatment recommendations across time points (T1 and T2) in control and experimental groups.

Interestingly, a significant group × time interaction was also found (F(1,87) = 16.03, *p* < 0.001, ηp^2^ = 0.16). Medical students in the experimental group were more likely to recommend therapy for the stimuli at T2 compared to T1 (T1: *M* = 4.71, SE = 0.19; T2: *M* = 5.70, SE = 0.18; *p* < 0.001). In contrast, no difference in treatment recommendations was observed between T1 and T2 in the control group (T1: *M* = 5.31, SE = 0.21; T2: *M* = 5.24, SE = 0.20; *p* = 0.72). See Figure 6. No other significant main effects or interactions emerged.

## 6 Discussion

The present study aimed to evaluate the effectiveness of a multifaceted educational intervention designed to mitigate biases related to race and trustworthiness inferences drawn from facial appearance in pain perception among medical students. Given the critical role that healthcare professionals play in diagnosing and managing pain, it is essential to enhance awareness and reduce such biases through targeted training. This approach fosters equitable and accurate pain management across diverse patient populations. Addressing implicit biases early in medical training is crucial for improving clinicians' ability to recognize and respond to pain in their patients, ultimately leading to enhanced patient care and improved health outcomes.

The results revealed that participating in the experience has influenced students regardless of their assigned group (control vs. experimental).

Completing the IAT at T1, even without receiving feedback on their final score, may have contributed to increase participants' awareness of bias and thereby contributed to improvement in both groups, including the control group. This potential effect aligns with literature, which highlights that engaging with the IAT can prompt self‐reflection and increase consciousness about implicit biases (Strand et al. 2021; van Ryn et al. 2015). Also, repeated exposure to the pain recognition task itself might influence behavioural responses, with participants becoming familiar with the task independently of their group and the time of educational intervention. This familiarity was influenced by both the race and facial trustworthiness of the stimuli. Indeed, a reduction has been found in race‐related differences in pain perception among medical students for both experimental and control groups. Specifically, medical students presented enhanced sensitivity to Black stimuli at T2 relative to T1 (i.e., faster response time to identify painful expressions, higher perceived pain ratings, and higher likelihood of recommending treatment). This effect aligns with existing explanations for mere exposure effects, which attribute the phenomenon to factors such as reduced uncertainty, enhanced perceptual fluency, and diminished negative affect toward a novel stimulus (Bornstein and Craver‐Lemley 2022). Additionally, the current findings specific for Black (i.e., other‐race) faces support the familiar face overgeneralization (FFO) hypothesis, which posits that racial bias partially stems from negative reactions to faces that diverge from the familiar own‐race prototype (Zebrowitz and Collins 1997), and that increasing familiarity through mere exposure can help mitigate this bias (Zebrowitz and Montepare 2008). In our study, the improvement in pain perception for Black stimuli could stem from participants becoming more attuned to subtle racial cues within the stimuli as a result of task repetition. Interestingly, the lack of improvement for White stimuli at T2 implies that the learning effect was asymmetrical. This asymmetry could reflect a dual process where familiarity heightens sensitivity to previously underperceived stimuli, in this case, pain in Black faces, while maintaining status quo biases toward more familiar White faces.

However, it is important to note that facial trustworthiness adds an important layer of complexity to the learning effect on perceived pain intensity and treatment recommendations. Even White faces, when looking trustworthy, benefit from the generalized improvement in sensitivity to pain cues, driven by task familiarization and growing attention to social cues as described above. This aligns with findings reporting enhanced empathic resonance with stimuli perceived as socially congruent or approachable (Azevedo et al. 2013; Drwecki et al. 2011). Conversely, the decrease in ratings on perceived pain intensity and treatment recommendations for untrustworthy‐looking White faces at T2 may reflect the interplay of cognitive dissonance and stereotype incongruence (Smith et al. 2017). In fact, although our stimuli were selected to ensure no differences in the level of facial trustworthiness between White and Black individuals, it is well established that race can influence trustworthiness judgments, with a tendency of perceiving White faces as more trustworthy (Charbonneau et al. 2020; Stanley et al. 2012). This occurs because people rely on a combination of observable social cues (e.g., race) and their own beliefs about these cues to form trustworthiness estimates (Stanley et al. 2012). Consequently, here, untrustworthy White faces may have represented incongruent stimuli, eliciting different responses to their painful expressions compared to other categories due to the mismatch between race‐based expectations and trustworthiness cues, particularly under conditions of repeated exposure.

Importantly, besides the not surprising effect of repeated exposure to the task, the educational intervention has been proved to be effective on students' response times, pain intensity ratings, and treatment recommendations across stimuli categories. In terms of response times, students in the control group became faster at T2 compared to T1, confirming the task‐familiarization effect. This improvement was absent in the experimental group, suggesting that the educational intervention might have heightened their awareness, effectively countering the automaticity that typically accompanies task repetition. This is in line with literature showing that bias awareness can moderate how people respond in a range of social situations, often improving the ability to make conscious, deliberate, and thus slower responses (Hahn and Gawronski 2019; Perry et al. 2015). Interestingly, the effects of the educational intervention extend beyond response times, with a different pattern in pain intensity ratings and treatment recommendations. Unlike the control group, students in the experimental group significantly increased their ratings and treatment recommendations from T1 to T2 across all categories of stimuli, regardless of race or facial trustworthiness, suggesting that the educational intervention effectively heightened their sensitivity to pain‐related cues and prompted a broader reassessment of their clinical judgments. These findings align with evidence showing that increasing knowledge about the science of implicit bias and increasing self‐awareness can recalibrate medical assessments (Ruben and Saks 2020; Sukhera and Watling 2018). It is important to note that the uniform increase across stimuli categories also indicates that the educational intervention not only influenced their responses to historically marginalized groups but generalized to all patient categories, promoting a more equitable approach to pain management.

## 7 Strengths and Limitations

Besides the measurement‐induced bias inherent to the study design, this study has some limitations that should be acknowledged. A limitation is the lack of follow‐up assessments to measure the long‐term impact of the multifaceted educational intervention. While a high percentage of students (80.22%) opted to report on this subject during their oral exams, indicating strong interest and immediate engagement, we cannot definitively ascertain whether the effects of the training are durable over time. Future research should investigate the lasting impact of such interventions by administering the pain recognition task after a significant interval to evaluate retention and applicability in their future clinical practice. Another limitation of the study is that all nine excluded participants were in the control group. This may limit the generalizability of the control group findings; however, it is important to note that this distribution appears to be coincidental, as group assignment was randomized and blinded, and no systematic cause was identified. Finally, we recognize that all students who participated in this intervention are White. This does not reflect the demographic composition of many medical universities globally. We do not have data on how beneficial this intervention would be for medical students who do not self‐identify as White or are in a more racially diverse medical program with peers that do not self‐identify as White. Future studies should repeat this research in a more diverse sample.

Despite these limitations, the study presents notable strengths. The educational intervention was designed to be engaging and easily implementable within existing curricula, making it a practical choice for busy academic programs. The positive feedback from students regarding the training further underscores its effectiveness and relevance. Additionally, the brief nature of the intervention demonstrates that focused, concise sessions can yield meaningful outcomes, suggesting a scalable approach that could be integrated into various healthcare training programs. This intervention that educates medical students to recognize pain‐related cues through facial expressions may be particularly valuable for improving pain treatment in individuals who are unable to verbally express their pain (Afenigus 2024).

However, it is important to note that the findings may not be fully generalizable across all healthcare disciplines. Differences in training programs and professional focuses, such as those between medical, nursing, and physiotherapy students, could influence both the perception of the intervention and its effectiveness. For instance, variations in diagnostic reasoning among different student groups (Pirret 2016) may lead to differing levels of engagement and impact.

## 8 Conclusion

To conclude, this study underscores critical educational and clinical implications, highlighting the importance of implementing effective educational training to address disparities in pain recognition and treatment that arise from facial cues. The findings are promising, indicating that even brief interventions can be effective in recognizing and managing implicit biases. This suggests significant potential for medical education to reduce physician contributions to healthcare disparities.

It is essential to meaningfully integrate these topics into healthcare professions curricula alongside medically focused content. Practical implementations might include courses that combine educational interventions aimed at increasing knowledge about implicit bias in healthcare with experiential simulations, such as implicit association tests (IAT) and behavioral tasks involving diverse patient profiles.

By equipping medical students with the skills to recognize and mitigate biases, we pave the way for enhanced awareness and more equitable treatment for diverse patient populations. Ongoing efforts to refine and assess the impact of these educational interventions will be essential in addressing the critical issue of pain treatment disparities within clinical practice.

## Author Contributions

This study was conceptualized and designed by A.B. and K.M. The experiment was performed by A.B. and I.C. The data were analysed by A.B., and the results were critically examined by all authors. A.B. had a primary role in preparing the manuscript, which was edited by I.C., F.S. and K.M. All authors have approved the final version of the manuscript and agree to be accountable for all aspects of the work.

## Funding

This study was supported by Mattarozzi RFO 2023.

## Conflicts of Interest

The authors declare no conflicts of interest.

## Supporting information


**Table S1:** Descriptive (M ± SD) of selected stimuli by race and trustworthiness on trustworthiness ratings.
**Table S2:** Descriptive (M ± SD) of selected stimuli by race and trustworthiness on pain ratings.
**Table S3:** Means (M) and standard deviations (SD) of trustworthiness ratings across groups.
**Table S4:** Means (M) and standard deviations (SD) of response times (in ms) across groups.
**Table S6:** Means (M) and standard deviations (SD) of treatment likelihood across groups.
