# Supplementary material for: Learning to Unveil: Tackling Implicit Bias in Pain Recognition Through Education
Source: Eur J Pain. 2026 Apr 25;30:e70281. doi: 10.1002/ejp.70281 (PMC13109812; doi:10.1002/ejp.70281)
Supplement: Supplementary file 1 — Table S1: Descriptive (M ± SD) of selected stimuli by race and trustworthiness on trustworthiness ratings. Table S2: Descriptive (M ± SD) of selected stimuli by race and trustworthiness on pain ratings. Table S3: Means (M) and standard deviations (SD) of trustworthiness ratings across groups. Table S4: Means (M) and standard deviations (SD) of response times (in ms) across groups. Table S6: Means (M) and standard deviations (SD) of treatment likelihood across groups. [file EJP-30-0-s001.docx]

**Supplementary Materials**

**Stimuli selection**

We selected faces categorized as White and Black from the Delaware Pain Database (DPD, Mende-Siedlecki et al., 2020) in order to have two separate stimuli sets, one for T1 and one for T2, ensuring no significant differences between the sets. The DPD includes pre-rated faces with established trustworthiness scores based on large normative samples. The selection process began by identifying faces with the highest and lowest trustworthiness ratings in the DPD, categorizing them as trustworthy-looking and untrustworthy-looking faces, respectively (T1: Trustworthy-looking faces M = 4.02, SD = .17, Untrustworthy-looking faces M = 2.80, SD = .32, *t*(14) = 9.60, *p* < .001; T2: Trustworthy-looking faces M = 4.00, SD = .25, Untrustworthy-looking faces M = 2.94, SD = .21, *t*(14) = 9.06, *p* < .001; T1 vs. T2: Trustworthiness × Time *F*_(1,32)_ = .97, *p* = .33, *η_p_^2^* = .03). We ensured that the chosen White and Black faces by each trustworthiness category did not differ significantly, within T1 stimuli set (T1: Race *F*_(1,16)_ = .04, *p* = .85, *η_p_^2^* = .003; Trustworthiness × Race *F*_(1,16)_ = .97, *p* = .48, *η_p_^2^* = .04) and T2 stimuli set (T2: Race *F*_(1,16)_ =3.34, *p* = .09, *η_p_^2^* = .21; Trustworthiness × Race *F*_(1,16)_ = 1.35, *p* = .27, *η_p_^2^* = .10), and between them (T1 vs T2: Trustworthiness × Time *F*_(1,32)_ = .98, *p* = .33, *η_p_^2^* = .04; Race × Time *F*_(1,32)_ = .97, *p* = .33, *η_p_^2^* = .04; Trustworthiness × Race × Time *F*_(1,32)_ = .21, *p* = .88, *η_p_^2^* = .001). See Table S1 for means and standard deviations.

**Table S1**

Descriptive (M ± SD) of selected stimuli by race and trustworthiness on trustworthiness ratings

|  | T1 | | T2 | |
| --- | --- | --- | --- | --- |
|  | White | Black | White | Black |
| Trustworthy-looking faces | 4.08 ± .19 | 3.95 ± .14 | 4.14 ± .23 | 3.83 ± .15 |
| Untrustworthy-looking faces | 2.76 ± .30 | 2.83 ± .38 | 2.98 ± .28 | 2.90 ± .17 |

Finally, we also verified that the selected stimuli did not differ in their pain ratings, as provided by the DPD, across race and trustworthiness categories, within T1 stimuli set (T1: Trustworthiness *F*_(1,16)_ = .61, *p* = .45, *η_p_^2^* = .05; Race *F*_(1,16)_ = 2.98, *p* = .11, *η_p_^2^* = .19; Trustworthiness × Race *F*_(1,16)_ = .17, *p* = .69, *η_p_^2^* = .01) and T2 stimuli set (T2: Trustworthiness *F*_(1,16)_ = .83, *p* = .38, *η_p_^2^* = .06; Race *F*_(1,16)_ = 1.86, *p* = .20, *η_p_^2^* = .13; Trustworthiness × Race *F*_(1,16)_ = .03, *p* = .87, *η_p_^2^* = .002)), and between them (T1 vs T2: Trustworthiness × Time *F*_(1,32)_ = .00, *p* = .98, *η_p_^2^* = .00; Race × Time *F*_(1,32)_ = .21, *p* = .65, *η_p_^2^* = .01; Trustworthiness × Race × Time *F*_(1,32)_ = .04, *p* = .84, *η_p_^2^* = .002). See Table S2 for means and standard deviations.

**Table S2**

Descriptive (M ± SD) of selected stimuli by race and trustworthiness on pain ratings

|  | T1 | | T2 | |
| --- | --- | --- | --- | --- |
|  | White | Black | White | Black |
| Trustworthy-looking faces | 4.18 ± .69 | 3.79 ± .18 | 4.11 ± .59 | 3.81 ± .36 |
| Untrustworthy-looking faces | 4.53 ± 84 | 3.89 ± .44 | 4.38 ± .33 | 3.99 ± .62 |

**Facial trustworthiness manipulation check**

The selected stimuli were perceived according to the trustworthiness categorization. An ANOVA on facial trustworthiness ratings at T1 revealed a significant main effect of facial trustworthiness (*F_(_*_1, 89)_ = 359.09, *p* < .001, η_p_^2^ = .80) and a significant interaction between facial trustworthiness and race (*F_(_*_1, 89)_ = 14.25, *p* < .001, η_p_^2^ = .14). Specifically, participants rated trustworthy faces as significantly more trustworthy than untrustworthy faces, regardless of whether the faces were White or Black. Importantly, the main effect of group (*F_(_*_1, 89)_ = 0.24, *p* = .63, η_p_^2^ = .003) and interactions between facial trustworthiness and group (*F_(_*_1, 89)_ = 0.25, *p* = .62, η_p_^2^ = .003) and between race and group (*F_(_*_1, 89)_ = 0.21, *p* = .65, η_p_^2^ = .002) were not significant, indicating no differences between the experimental and control groups in facial trustworthiness ratings across races before the educational intervention. Table S3 reports the means and standard deviations of facial trustworthiness ratings across groups.

**Table S3**

*Means (M) and standard deviations (SD) of trustworthiness ratings across groups*

|  |  | Total  (N=91) | | Experimental group (N=50) | | Control group  (N=41) | |
| --- | --- | --- | --- | --- | --- | --- | --- |
|  |  | *M* | *SD* | *M* | *SD* | *M* | *SD* |
| Trustworthy faces | White | 5.59 | 1.26 | 5.65 | 1.27 | 5.51 | 1.27 |
|  | Black | 5.27 | 1.46 | 5.36 | 1.27 | 5.18 | 1.66 |
| Untrustworthy faces | White | 4.02 | 1.31 | 4.10 | 1.33 | 3.92 | 1.29 |
|  | Black | 3.18 | 1.21 | 3.15 | 1.20 | 3.21 | 1.24 |

**Baseline equivalence check between the experimental and control groups**

*Response time*

Mixed ANOVA results showed no significant main effect of group, *F*(1,89) = 0.14, *p* = .71, *η_p_²* = .002, and no significant interactions involving group: group × facial trustworthiness, *F*_(1,89)_ = 1.40, *p* = 0.24, *η_p_²* = .01; group × race, *F*_(1,89)_ = 0.29, *p* = .59, *η_p_²* = .003; and group × facial trustworthiness × race, *F*_(1,89)_ = 0.59, *p* = .44, *η_p_²* = .007, indicating that response times were comparable between the experimental and control groups across all conditions at T1. Table S4 reports the means and standard deviations of response times across groups.

**Table S4**

*Means (M) and standard deviations (SD) of response times (in ms) across groups*

|  |  | Total  (N=91) | | Experimental group (N=50) | | Control group  (N=41) | |
| --- | --- | --- | --- | --- | --- | --- | --- |
|  |  | *M* | *SD* | *M* | *SD* | *M* | *SD* |
| Trustworthy faces | White | 4118.82 | 923.41 | 4088.37 | 787.05 | 4155.95 | 1075.95 |
|  | Black | 4304.12 | 911.81 | 4316.23 | 871.81 | 4289.34 | 969.91 |
| Untrustworthy faces | White | 3947.18 | 968.25 | 3898.70 | 903.72 | 4006.29 | 1049.97 |
|  | Black | 4340.94 | 916.51 | 4282.07 | 821.79 | 4412.72 | 1026.18 |

*Perceived pain intensity*

Mixed ANOVA results showed no significant main effect of group, *F*_(1,89)_ = 2.37, *p* = .13, *η_p_²* = .03, and no significant interactions involving group: group × facial trustworthiness, *F*_(1,89)_ = 0.42, *p* = .52, *η_p_²* = .005; group × race, *F*_(1,89)_ = 0.65, *p* = .42, *η_p_²* = .007; and group × facial trustworthiness × race, *F*_(1,89)_ = 0.00, *p* = .99, *η_p_²* = .000. These findings confirm that perceived pain intensity was comparable between the experimental and control groups across all conditions at T1. Table S5 reports the means and standard deviations of pain intensity ratings across groups.

*Treatment recommendation*

Mixed ANOVA results showed no significant main effect of group, *F*_(1,89)_ = 3.60, *p* = .08, *η_p_²* = .04, and no significant interactions involving group: group × facial trustworthiness, *F*_(1,89)_ = 1.21, *p* = .27, *η_p_²* = .01; group × race, *F*_(1,89)_ = 0.39, *p* = .53, *η_p_²* = .004; and group × facial trustworthiness × race, *F*_(1,89)_ = 1.86, *p* = .18, *η_p_²* = .02. These results indicate that the likelihood of recommending treatment was comparable between the experimental and control groups across all conditions at T1. Table S6 reports the means and standard deviations of treatment recommendation across groups.

**Table S6**

*Means (M) and standard deviations (SD) of treatment likelihood across groups*

|  |  | Total  (N=91) | | Experimental group (N=50) | | Control group  (N=41) | |
| --- | --- | --- | --- | --- | --- | --- | --- |
|  |  | *M* | *SD* | *M* | *SD* | *M* | *SD* |
| Trustworthy faces | White | 5.11 | 1.56 | 4.99 | 1.56 | 5.26 | 1.58 |
|  | Black | 4.40 | 1.54 | 4.13 | 1.43 | 4.73 | 1.61 |
| Untrustworthy faces | White | 5.34 | 1.59 | 5.01 | 1.56 | 5.74 | 1.42 |
|  | Black | 5.17 | 1.61 | 4.91 | 1.61 | 5.50 | 1.58 |
